# Hospital and Institutional News

**Published:** 1916-12-09

**Authors:** 


### HOSPITAL AND INSTITUTIONAL NEWS.

### A NEW APPOINTMENT AT GUY'S HOSPITAL.

THE authorities of Guy's Hospital are advertising a new post in connection with the treatment of venereal diseases, and the wording of the announcement suggests an interesting point for hospital managers in connection with the new facilities which are to be provided under the Local Government Board schemes for the treatment of these diseases. The question at issue, or that may be at issue, is whether departments working such schemes should be under specialists appointed *ad hoc* and ranking with the other special visiting staffs; whether they should be under the dermatological specialists whom all large Metropolitan hospitals possess; or whether (as far as the female patients are concerned) they should be under the gynæcologists. Both the two former propositions have supporters; and, although the arguments in favour of the first plan seem to us the more cogent, there is certainly something to be said for the second of the two. It is difficult to comprehend why the third plan should commend itself to the authorities at Guy's Hospital, as it appears to have done. Venereal diseases are no monopoly of the female sex, nor when they affect women do they involve solely, or necessarily at all, the special pelvic organs of that sex; nor have gynæcologists, as a class, shown any special aptitude for the treatment or the prevention of venereal diseases; nor is there any reason why the treatment of these diseases in the male should be in other hands than is that of the same diseases in the female. It will be interesting to note whether other hospitals follow the lead of Guy's; and some form of conference to decide on a uniform principle of action and to thresh out relative advantages and disadvantages should, in our opinion, be held forthwith, if it is not already too late.

### TETANUS IN HOME MILITARY HOSPITALS.

IN a review of the cases of tetanus occurring in home military hospitals between August 1, 1915, and July 31, 1916, Surgeon-General Sir David Bruce summarises his views on the lessons taught by experience during what may roughly be termed the second year of the war. Those who know Sir D. Bruce's high reputation as a scientist will note the caution with which he draws his conclusions, largely because the comparatively small number of cases (195) reported invalidates to some degree the statistical results. They may also regret that Sir David has not been able to include the tetanus cases treated abroad (at least in the B.E.F.), and that he makes so little effort to assess the value of the prophylactic inoculation of wounded soldiers with anti-tetanic serum. The former omission spoils the results, because thereby most of the very serious cases, which never reach home, are excluded; and the latter is regrettable because every piece of additional evidence on the point is valuable, notwithstanding that the overwhelming mass of that already collected supports the advocates of this method of preventive treatment. The most important of Sir D. Bruce's findings is that the evidence about the therapeutic (as opposed to the preventive) value of anti-tetanic serum is still inconclusive: though we note that the statistics do show a distinctly smaller mortality when it is used, and Sir David continues to recommend its usage in all cases of developed tetanus. He also perceives no special advantage in the intrathecal route of administration over subcutaneous or other injections. He drily remarks that there are no allusions to the use of anti-tetanic serum as a prophylactic in home military hospitals, though he abstains from an expression of opinion as to whether such injections are desirable. The one point affecting the medical officers of home military hospitals upon which Sir D. Bruce takes a definitely helpful line is as to whether the surgeon should or should not explore the wound and lay it freely open as part of the treatment of tetanus. Preventively he is strongly in favour of such procedures; but when tetanus has actually supervened he distinctly deprecates them.

### A NICE POINT FOR INFIRMARY MANAGERS.

ON a recent occasion a dock labourer was summoned before the Stratford Police Court for the cost of his child's keep in the West Ham Union Infirmary, to which it had been admitted at his request. The Guardians discovered that the defendant earned three pounds a week and his wife two pounds, and sued for twenty-four shillings and sixpence weekly as the cost of the baby's treatment. The chairman of the magistrates took up the cudgels with some warmth on behalf of the dock labourer, maintaining that for the cost of a baby's keep the sums demanded were preposterous. He elicited from the official who appeared in support of the summons that the figure was arrived at by averaging the total cost of the infirmary against the number of patients under treatment; and that salaries of officials were included in the expenses of the infirmary estimated for this purpose. He appears to have cast ridicule on this method of calculation on the ground that a baby eats much less than an adult, though he did not suggest an alternative plan of apportionment. Nor did he, apparently, take into consideration the fact that the baby is ill, and therefore requires nursing and medical attendance. All things considered, it is difficult to see that the sum claimed is excessive as the cost of treatment of a sick baby; and the suggestion that establishment charges should be disassociated from the recoverable expense due to the ratepayers in respect of the child of parents earning £260 a year strikes us as indefensible.

### STATE ENCOURAGEMENT OF RESEARCH.

THE Government have decided to establish a separate Department of Scientific and Industrial Research for Great Britain and Ireland, under the Lord President of the Council, with the President of the Board of Education as Vice-President. They have also decided, subject to the consent of Parliament, to place a large sum of money at the disposal of the new Department, to be used as a fund for the conduct of research for the benefit of the national industries on a co-operative basis. The Board of Inland Revenue have decided, with the approval of the Chancellor of the Exchequer, that no objection shall be offered by their surveyors of taxes to the allowance, as a working expense for income-tax purposes, of contributions by traders to industrial associations which may be formed for the sole purpose of scientific research for the benefit of the various trades; and the allowance would be equally applicable as regards traders' contributions specifically earmarked to the sole purpose of the Research Section of an adapted existing Association. In both cases the allowance would be subject to certain conditions—*e.g.* the Association or the Research Section to be under Government supervision and the traders' contribution to be an out-and-out payment, made from his trade profits and giving him no proprietary interest in the property of the Association, etc. In order to enable the Department to hold the new fund and any other money or property for research purposes, a Royal Charter has been granted to the official members of the Committee of the Privy Council for Scientific and Industrial Research under the title of the "Imperial Trust for the Encouragement of Scientific and Industrial Research." The Trust is empowered "to accept, hold, and dispose of money or other personal property in furtherance of the objects for which it has been established, including sums voted by Parliament to that end." The Trust can take and hold land and can "accept any Trusts, whether subject to special conditions or not, in furtherance of the said objects." A substantial gift has already been made to the Trust by two members of the Institution of Mechanical Engineers for the conduct of a research in mechanical engineering to be approved by the Department, in the hope that this example will be followed by other members of the Institution.

### THE LATEST DESIGN FOR AN OPEN-AIR SCHOOL.

The Glasgow School Board, which claims to be the pioneer of properly designed open-air schools for defective children, has taken a further step in opening a specially constructed school for the normal child. Known as the Bernard Street Public School, the new institution, which is interesting in design and construction, has been planned by Mr. David Maben, the School Board's Master of Works. On a site of 8,071 square yards an E-shaped building, two storeys in height, provides two separate entrances for boys and girls. There are twenty-two ordinary class-rooms, each of which accommodates forty children, and gives 15 square feet to each child. In special rooms art, manual work, sewing, housewifery, and so on are taught. A special unit provides for the medical inspection of the scholars: a noteworthy accession. There are also a gymnasium, a drill-hall, and last, but luckily, a library. In what may be called the bath block a series of separate bathing and dressing cubicles has been provided, together with twenty spray baths. The construction is of "brick built in cement," with stone facings. Each classroom has four north windows, a borrowed light on the south, which is furnished with a loggia, while the entrance is by a sliding door 8 feet wide, which, when open, provides an open-air class-room. Double-hung windows, communication between the blocks by outside corridors, open at one side, garden spaces in the playground—these indicate the care spent on the new open-air school. The Glasgow School Board are to be congratulated on their far-sighted and ambitious scheme.

### EXTENSIONS AT KING EDWARD VII.'s HOSPITAL, CARDIFF.

In spite of difficulties, the scheme for the extension of King Edward VII.'s Hospital continues to be pushed forward by the tireless energies of the committee and supporters of the institution. We may add to our recent statements on the subject that, in addition to Sir Edward Nicholl's gift of £50,000 for a nurses' home for 160 nurses, and a maternity hospital for eighty-nine beds, Colonel Bruce Vaughan has made a conditional promise to take early steps to reduce the waiting-list. He lately stated that, if no interference occurred from the Government, within eight months he hoped that ninety beds would be added to the hospital. This, it will be seen, would be a definite and early extension; and so long as the money is given, the plans laid, and the labour question provided for, as has been the case with the King Edward's Hospital schemes, we can hardly believe that any Government can persist in damming the stream and blocking the way for ever.

### IN PLACE OF THE ANNUAL DINNER.

The meeting held on Thursday (December 7) at the Drapers' Hall, Throgmorton Street, on behalf of the Poplar Hospital, is an instance of the means adopted in war-time to find a substitute for the annual festival dinner on which some institutions have been accustomed to rely for a large part of their income. The Poplar Hospital for Accidents has been so closely associated with the Drapers' Company, to which it owes many generous gifts, that it may be counted fortunate in being able to arrange for such a historic place of meeting. Last year, if we remember, a similar meeting was held at The Baltic, another invaluable centre on which the hospital had, as it were, a claim owing to its work in connection with the docks. We have previously emphasised the value of such meetings when permission can be gained to hold them in the halls of the great City Companies and other places with a distinction of their own. But hospitals with no "claims" are hard put to it to find a suitable substitute.

### KIRKCALDY HOSPITAL ND THE NAIRN FAMILY.

The latest extension to Kirkcaldy Hospital, which has been opened by Sir Michael Nairn, once again illustrates the part played by single families in the history of Scotch, as well as English, hospitals. In an interesting speech the opener recalled how twenty-six years ago the original section of the hospital had been opened as his father's gift. The building then comprised ten beds, four cots, a theatre, and staff quarters. In 1898 this accommodation was doubled. Once again the need for extension arose, and his father, by doubling the accommodation, made the hospital four times its original size. It now includes a new theatre, an $x$-ray installation, and also two private wards. The present baronet, by opening the new extension, carries on the work which his family has made its own.

### THE IRISH LOCAL GOVERNMENT BOARD AND DISLOYAL BOARDS OF GUARDIANS.

The attempts of the Irish Local Government Board to promote the recruiting of medical officers for the R.A.M.C. by refusing to sanction appointments of practitioners under forty-one years of age to posts under Boards of Guardians in Ireland have already been mentioned on two former occasions in these columns (The Hospital, September 9, 1916, p. 536; and September 23, 1916, p. 577). The latest development of this attempt to counteract by bureaucratic methods in one small sphere the active disloyalty to the Allied cause of the greater part of Ireland is to be seen in the continued contumacy of the Letterkenny Guardians, who have taken counsel's opinion as to whether the Local Government Board is within its rights in refusing to sanction one such appointment, that of a young Irish doctor of twenty-one years of age as medical officer to the Guardians. An Irish M.P., who is also a King's Counsel, has advised the Guardians that the Local Government Board is exceeding its powers. Whether he is right, the Courts will doubtless have to decide. Meanwhile it might be worth while for every English, Scottish, and Welsh hospital, Board of Guardians, County Council, Borough, and other authority employing medical men to pass a resolution debarring from candidature, both during and after the war, all qualified practitioners who may have been at any period of the war less than forty-one years of age and have not served in the Army or been medically rejected as unfit for service. It is true that such a restriction would probably have little effect upon disaffected young Irish doctors at the present time. But it would at least be fair to the other three nationalities and would also exclude the conscientious objector and other skulkers and shirkers.

### THE CANADIAN DISCHARGE DEPOT AT BUXTON.

The Canadian Discharge Depôt, which has been recently at Bath and Shoreham, has now been transferred to Buxton. The Empire Hotel, which has been taken by the Imperial authorities for this purpose, is magnificently situated and enclosed by spacious grounds. All Canadian soldiers who have been treated in various hospitals in England and are fit to be moved will be sent to Buxton, whence they will be transferred to Canada for further treatment or for discharge when a final decision has been made at the depôt. Buxton has been chosen for this purpose owing to its splendid situation and its exhilarating climate, and on account of its being convenient for embarkation. The number of men to be dealt with at a time will be about 1,500, and therefore a very large staff will have to be kept. Lieut.-Colonel Paul R. Hanson is the commanding officer, and Major C. G. Arthur, D.S.O., is second in command. About six months ago the Canadian military authorities took over the Peak Hydro Hotel, Buxton, and converted it into a large special hospital of 300 beds for soldiers suffering from various forms of rheumatism, nervous diseases, and shell shock contracted whilst serving with the Canadian Expeditionary Forces. The building has been beautifully fitted up and equipped; nor has expense been spared to make the place comfortable for the patients. All manner of electrical treatments and massage are given at the hospital, whilst the town authorities have made special arrangements to give the soldiers mineral-water treatments at the public thermal bathing establishments.

### THE DERBY ROYAL INFIRMARY.

For over a century the Derby Royal Infirmary has always found generous support, but it has rarely, if ever, been able to show a surplus of £100 over expenditure, as it does this year. At one time the management feared a deficit of £2,000 or £3,000. The income, however, more than kept pace with the increasing cost of food and other commodities, so that, although the expenditure went up to £2,579 more than in the previous year, the financial position of the institution is more favourable than it has been for a long time past. Sir Thomas Roe, M.P., has succeeded to the presidency in the place of the Duke of Devonshire, whose services for the last seven years have been recognised by resolution.

### A NORTH OF ENGLAND HOSPITAL FOR INCURABLES.

A total deficit for the year of £456 was reported at the annual meeting in Manchester last week of the Northern Counties Hospital for Incurables—which has homes at Mauldeth and Walmersley, near Bury—and Mr. Alfred Crewdson, the chairman, made an earnest appeal for increased subscriptions. The hospital now shelters 114 patients. Mr. Crewdson, Mr. R. G. Lawson, and Mr. G. Rhodes, K.C., have been reappointed members of the board.

### FOOD PRICES AT ST. BARTHOLOMEW'S.

The public likes to read about everyday things, and there is no doubt that a hospital like St. Bartholomew's, that publishes from time to time particulars of its food prices, obtains a good advertisement of its needs. A recent report of the great City hospital shows that for the first six months of the current year there was an increased cost of £2,200 under the heading of provisions alone. With the exception of milk, no contracts had been entered into and all food was bought at prevailing market prices. The following articles have practically doubled in cost as against the period of six months immediately before the war:—Beef, legs of mutton, butter, bread, and flour; suet is 11d. per lb., as against 4d.; eggs are more than three times dearer, and sugar shows an increase of about 150 per cent. There has been no change in the dietary of the patients. There is no mention of margarine in the list of comestibles, but butter is bought at a shade under 2s. per lb. If the patients and staff are given butter they are afforded a luxury which, judging by general experience, is probably absent from the tables of many of the subscribers to hospitals. To spend charitable funds on butter when there is such an excellent substitute is an act which is not easily justified.

### LEISURELY PROGRESS IN THE GOWRIE HOUSE DISPUTE.

The extraordinary *impasse* in connection with the Gowrie House Asylum scandal at Dundee has already been described in these columns (see The Hospital, November 4, 1916, p. 80; and November 18, 1916, p. 133). On the latter occasion the opinion was expressed that the Scottish Board of Control was not likely to submit to be openly flouted by the local Parish Council; and that as its wishes were being disregarded and its plain hints ignored, more was likely to be heard of the matter. Our prognostications have turned out to be accurate, for the Board of Control is taking a distinctly firmer line, though still not firm enough to satisfy some of the most ardent reformers in the Dundee local press. The Board of Control has sent a letter to the Parish Council controverting many of the statements in the letter which was written by the latter in defence of its refusal to remove the medical superintendent. The most important new fact now enunciated by the Board is that this official has already in previous years been the subject of severe adverse criticism, and that his recent shortcomings are by no means isolated instances. The Board clearly regards it as essential that he shall be removed from office, with which view, for reasons given in our issue of November 18, we feel bound to concur. The Parish Council would take no notice of the velvet glove with which the Board of Control has been handling the matter, but the iron hand is there all the same; and compliance with the Board's suggestions is undoubtedly the right policy if serious trouble is to be avoided.

### THE CARNARVONSHIRE AND ANGLESEY INFIRMARY.

A forward movement has been decided upon in connection with the Carnarvonshire and Anglesey Infirmary, of which Lord Penrhyn has been re-elected president. At the annual meeting of the governors last week the following resolution was adopted, after discussion: "That this meeting recommends that the general committee take early steps to establish a building fund for the erection of a new wing, and for this purpose to issue an appeal." Emphasis was laid by the medical staff upon the urgent necessity of meeting the ever-increasing demands upon the institution, and the general committee, in its report, expressed the opinion that a well-equipped new wing would form a most fitting memorial to the brave men from the two titular counties who have died for their country. The nucleus of a building fund has been formed, but it is suggested that the scheme cannot very well be launched in war-time. The governors, however, think it should be proceeded with, and Dr. Thomas urged the establishment at the same time of a training-school for nurses, with power to issue certificates, and also the establishment of a nursing home in connection with the institution.

### A NEW CONVALESCENT HOSPITAL AT HARROGATE.

Lord Furness has offered, subject to the approval of the shareholders, who are to be met on a business footing, to bear the entire cost of transforming the Grand Hotel, Harrogate, into a convalescent home for officers, and himself maintain it as such for the duration of the war. If Lord Furness's generous idea is carried out, it is intended to open the hotel as an institution early in the New Year, and to provide accommodation there for 200 officers. The Grand is one of the best-known hotels at Harrogate. It is close to the bathing establishments, and overlooks the Valley Gardens.

### THIS WEEK'S DRUG MARKET.

Few noteworthy price changes have occurred since our last report was written. Of late values have been much steadier, and violent fluctuations of the kind that were of common occurrence some time ago are now rarely witnessed. The constant increase in freights is, of course, bound to affect the prices of drugs that come from overseas—as most drugs do—and all bulky articles of this kind have an upward tendency in value; should the shortage of ships become more acute, this condition will become more pronounced. Synthetic drugs have for the most part reached a level that does not allow of sudden and substantial downward movements; take, for instance, the case of salicylate of soda—the value is now about one-third the figure that was touched at one time, and although the output of this product is steadily declining, it is obvious that any further reductions must be gradual and small. Phenacetin still fetches very high prices; this is one of the synthetic drugs that is not being manufactured in this country—so far as can be assertained—on any important scale; the main reason for this is that somewhat elaborate plant is required, and manufacturers are so fully engaged in other directions that they hesitate to tackle a new venture of this kind. In America, whence we obtain much of our supply of phenacetin, higher prices are ruling than here, American holders of this now costly substance holding out for big profits. Quinine is a quiet market, with prices tending downwards—but not rapidly. Potassium permanganate has become still scarcer, and this week even higher prices have been paid than last week; here we have an article which before the war was freely sold by the ton and now is doled out by the pound—it is something like twenty times its pre-war value. Quicksilver is dearer, and the salts of mercury may advance in price accordingly.

